# State-of-the-Art Management of Complications of Myeloma and Its Treatment

**DOI:** 10.1155/2010/343089

**Published:** 2010-06-27

**Authors:** Monique A. Hartley-Brown, Daniel M. Sullivan, Rachid Baz

**Affiliations:** ^1^Blood and Marrow Transplant Department, H. Lee Moffitt Cancer Center and Research Institute, 12902 Magnolia Dr, Tampa, FL 33612, USA; ^2^Department of Malignant Hematology, H. Lee Moffitt Cancer Center and Research Institute, 12902 Magnolia Dr, Tampa, FL 33612, USA

## Abstract

Multiple myeloma is an incurable disease, although patient survival has increased with the availability of novel agents. Both multiple myeloma and its therapies often affect the renal, immune, skeletal, hematologic, and nervous systems. The resulting organ dysfunctions often impair the quality of life of affected patients, complicate and limit subsequent therapies, and may result in significant mortality. Research on the treatment of complications of multiple myeloma has been limited; hence, preventative and management strategies for patients with these complications are heterogeneous and often based on anecdotal experience. In this paper, we review the effects of myeloma and the novel therapies on organ systems and suggest management strategies.

## 1. Introduction

The past decade has witnessed significant advances in the treatment of multiple myeloma with the introduction of high-dose therapy and stem cell transplantation into routine clinical practice, as well as the approval by the Food and Drug Administration (FDA) of four therapeutic agents, namely, lenalidomide, thalidomide, bortezomib, and pegylated liposomal doxorubicin, with each demonstrating survival benefit in patients with the disease [1–4]. Despite the improved survival of patients, the disease remains uniformly fatal [5]. In addition, the era of novel agents has been marked by the emergence of newer toxicities and complications associated with therapy and long-term survivorship. These complications frequently diminish the quality of life of patients, complicate and limit further therapy for the disease, and can result in mortality. Little research has been performed in this area, and many of the recommendations have been historically based on anecdotal data and expert opinions. 

 This review article focuses on five organ systems most commonly affected by multiple myeloma and its treatments, namely, the renal, immune, thromboembolic, skeletal, and peripheral nervous systems. In addition, we discuss management strategies to improve upon supportive therapies in treating patients with multiple myeloma.

## 2. Renal Dysfunction: Etiologies and Management

Renal failure is a frequent finding in patients with multiple myeloma, affecting as many as 50% of patients during the course of the disease and approximately 20% at diagnosis [6]. Renal failure can be secondary to the myeloma paraprotein (such as in cast nephropathy, amyloidosis, and light chain deposition disease) or related to complications of the disease (hypercalcemia, secondary to often used drugs such as bisphosphonates, nonsteroidal antiinflammatory drugs, intravenous contrast, or aminoglycosides, or prerenal azotemia) [7]. Nonparaprotein causes of renal insufficiency are not discussed here and are beyond the scope of this review. Accordingly, the etiology of renal failure in this setting may be difficult to establish, but kidney biopsy could sometimes be helpful in delineating the future care of these patients. 

Cast nephropathy is the most frequent cause of paraprotein renal disease in patients with myeloma, accounting for two-thirds of those with this disease [8]. Cast formation usually occurs in the distal nephron due to the precipitation of light chain with Tamm-Horsfall proteins. This results in damage to the renal epithelium, allowing passage of the light chains into the interstitium and causing inflammation and fibrosis [9]. Additionally, endocytosis of the light chains in the proximal tubules triggers activation of nuclear factor-*κ*B, (NF-*κ*B), which in turn leads to the synthesis and activation of inflammatory signaling pathways such as mitogen-activated protein kinase, extracellular signal-related kinase, and Jun N-terminal kinase pathways, all causing further inflammation and fibrosis [10]. One group observed that a vasoactive intestinal peptide could inhibit this process. These researchers showed that this peptide inhibited light chain-induced renal production of IL-6 and TNF-*α* in rats and light chain-induced renal epithelial injury in vitro, thus suggesting possible future application for myeloma renal disease [11]. In rare cases, crystal deposition in the tubules can result in severe and rapid renal failure, a process known as “crystal nephropathy,” which portends a poorer prognosis [8, 12]. Current therapy for cast nephropathy involves treatment of the underlying myeloma with or without plasmapheresis, which can often lead to reversal of cast nephropathy if therapy is instituted early [13]. The role of plasmapheresis in cast nephropathy is controversial. Two small single institution studies randomized patients with biopsy-proven cast nephropathy, and noted a significant improvement in renal disease (and in survival in one study) with plasmapheresis [13, 14]. A larger multi-center Canadian study suggested no significant improvement in renal function with plasmapheresis in an unselected group of myeloma patients with renal failure [15]. The European trial of free light chain removal by extended hemodialysis (EuLITE) is currently investigating the benefit of removing circulating free light chains by hemodialysis in patients with cast nephropathy using two Gambro HCO 1100 dialyzers in series [16]. 

 Primary systemic amyloidosis represents another cause of renal dysfunction in patients with myeloma. In renal amyloidosis, the immunoglobulin light chain fibrils are deposited in the mesangium of kidneys, resulting in proteinuria and nephrotic syndrome [17]. The diagnosis of amyloidosis requires the demonstration of apple green birefringence on Congo red staining of involved tissue. Immunostain for kappa or lambda light chain and electron microscopy are important adjunctive confirmatory tests. Systemic amyloidosis can often be diagnosed by a less invasive method via fat pad biopsy. Patients with concomitant primary (AL) amyloidosis and myeloma tend to have poorer prognosis, due to the additional adverse effects that AL amyloidosis may have on other major organs such as the heart, nervous system, gastrointestinal system, and kidneys [18]. Treatment of renal amyloidosis is aimed at controlling the plasma cell disorder using chemotherapy, targeted agents, and stem cell transplantation as indicated [19–21]. 

 Light chain deposition disease, first described in 1976 by Randall et al. [22], involves deposition of monoclonal, amorphous, nonCongophilic light chain immunoglobulins in various organs in the body, most commonly the kidneys. The rates of incidence of this disease in autopsies of patients with myeloma range from 5% to 11% [23]. The majority of patients have kappa light chain deposition. Although most patients with this disease have an identifiable monoclonal protein in the serum and/or urine, 25% of patients have no detectable monoclonal light chain by standard testing, and free light chain assays may be helpful [24]. Renal involvement results in renal insufficiency, proteinuria, and nephrotic syndrome from the light chain deposits in the mesangium, thereby forming nodular lesions (that may mimic diabetic nephropathy under light microscopy). In addition, associated tubular thickening or atrophy may also be noted. Immunofluorescence helps to differentiate these nodular lesions from diabetic lesions, but renal biopsy is required for diagnosis [25, 26]. The goal of therapy involves treating the underlying plasma cell dyscrasia. Often, renal recovery is not seen. For patients who have severe renal failure, renal transplantation can be considered, although few reports support this practice. 

 In summary, identifying the etiology of renal dysfunction in patients with multiple myeloma can be difficult but is of importance and often steers management decisions.

## 3. Infectious Complications and Prophylaxis

Multiple myeloma results in an immunodeficient state with dysfunctions in both the cellular and the humoral immune system. Hypogammaglobulinemia, T-cell dysfunction, and granulocytopenia (secondary to marrow involvement and therapy) result in increased infectious complications, with bacterial infections (encapsulated organisms) being the most common and resulting in a high mortality and morbidity. Bacterial infections are twice as likely during active therapy and during induction therapy (a rate of 3 infections per year compared to 1.5 infections per year, resp.) [27]. In addition, treatment with high-dose dexamethasone-based regimens is associated with a higher rate of severe infections and induction death compared to regimens containing lower dose dexamethasone (16% versus 7%, resp.) [28]. Primary prophylaxis with antibiotics is occasionally recommended, depending on the intensity of the induction regimen. In one study, a significant reduction in infectious complications (absolute rate of reduction of 88%) occurred with the use of trimethoprim-sulfamethoxazole for the first 2 months of induction chemotherapy [27]. Fluoroquinolones are other commonly used prophylactic antibiotics with some multi-agent induction regimens. Routine use of antifungal agents with commonly prescribed induction regimens are not recommended because of the relatively low incidence of invasive fungal infections despite corticosteroid therapy. Varicella zoster virus reactivation has been reported in bortezomib-treated patients, although routine use of acyclovir or valacyclovir (at prophylactic doses) has been found to be effective at reducing this risk [3, 29]. 

Another prophylactic strategy involves the use of intravenous immunoglobulins in patients with hypogammaglobulinemia and recurrent infections. This practice is supported by a randomized controlled clinical trial in 82 patients with stable multiple myeloma, comparing intravenous immunoglobulins (0.4 g/kg monthly for 1 year) to placebo. This trial noted no episode of pneumonia and septicemia in the treatment group compared to 10 such episodes in the placebo group [30]. 

 Vaccine-induced specific immunity is suspected to be unreliable in myeloma patients. Because of the lack of studies in this patient population, there are no specific recommendations and data regarding vaccination effectiveness in multiple myeloma patients. Despite reports suggesting a decreased immunogenicity [31] of pneumococcal and influenza vaccines in multiple myeloma patients, the risk-to-benefit ratio favors recommending these vaccines, although monitoring of postvaccination titers is generally not recommended. Recently, one study noted a higher effectiveness of pneumococcal vaccination in multiple myeloma patients treated with lenalidomide (an immune modulator) [32]. In 2008, the FDA approved the use of a varicella zoster virus vaccine in patients older than 60 years of age; however, this live attenuated vaccine is not recommended in immunocompromised patients, including patients with multiple myeloma [33, 34].

## 4. Medical and Surgical Management of Bone Disease

**Figure 1:**
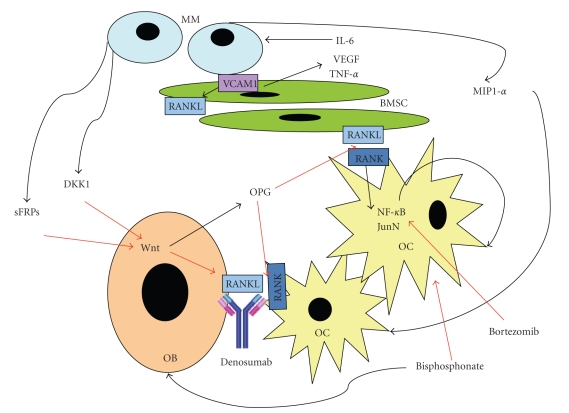
Pathophysiology of myeloma bone disease. Black arrows indicate increased production or activation, and red arrows indicate decreased production or inhibition. Normal bone metabolism involves the constant rebuilding of bone by osteoblasts and resorption of bone by osteoclasts, intricately balanced through appropriate cytokine signaling (usually associated with an increased OPG to RANKL ratio). The presence of myeloma cells in the bone marrow milieu results in upregulation of osteoclastic activity compared with osteoblastic bone formation resulting in lytic lesions. The malignant plasma cells interact with stromal cells, causing increased cytokine signaling through IL-6 and TNF-*β*. This results in increased RANKL expression by bone marrow stromal cells, stimulation of plasma cell growth, and drug resistance. In addition, malignant plasma cells produce inhibitors of Wnt signaling (DKK1 and sFRPs). Wnt inhibition results in downregulation of OPG production and upregulation of RANKL. The interaction of RANK with its ligand (RANKL) leads to osteoclast activation via signaling by NF-*κ*B. In addition, malignant plasma cells produce MIP-1*α*, which directly stimulates osteoclasts. Therapy with bisphosphonates results in osteoclast inhibition and osteoblast activation. The novel monoclonal antibody, denosumab, which neutralizes RANKL, is currently the subject of clinical investigations for bone disease therapy in patients with multiple myeloma. MM: multiple myeloma, IL-6: interleukin 6, VEGF: vascular endothelial growth factor, TNF-*α*: tumor necrosis factor-*α*, BMSC: bone marrow stromal cell, MIP-1*α*: macrophage inhibitory protein-1*α*, OB: osteoblast, OC: osteoclasts, OPG: osteoprotegerin, DKK1: dickkopf, sFRPs: serum frizzle related proteins, VCAM-1: vascular cell adhesion molecule-1, RANK: receptor activator for nuclear factor *κ*B, JunN: c-Jun N-terminal protein kinase, Wnt, Wnt glycoproteins (bind to sFRPs).

Myeloma bone disease is characterized by an increase in bone resorption over bone formation, resulting in lytic lesions and osteoporosis. Thus, patients have a propensity for fractures (most commonly in the vertebral spine). The pathophysiology of myeloma bone disease involves the receptor activator nuclear factor-*κ*B (RANK), which controls osteoclastic activation. The RANK ligand (RANKL) and a regulatory protein, osteoprotegerin, produced by stromal cells both bind RANK. Osteoprotegerin is a mimic for RANKL and inhibits signaling of the RANK-RANKL complex. Myeloma cells upregulate RANKL and downregulate osteoprotegerin, resulting in increased osteoclastic activity. This upregulation occurs through cytokine signaling of IL-6, TNF-*β*, and insulin-like growth factor (IGF), all of which are increased due to interaction of myeloma cells with stromal cells (Figure 1). In addition, DKK1, an inhibitor of Wnt, has been found to be important in osteoblastic cell growth and differentiation. Monoclonal antibodies targeting DKK1 are currently the subject of investigations [35].

 To determine the severity of disease and to identify complications during and before treatment, assessment of bony involvement is required at diagnosis. Up to 80% of myeloma patients at diagnosis are noted to have abnormalities on plain radiographs. This is usually in the form of punched-out lytic lesions, but vertebral compression fractures are also noted in about half of the patients [36]. A baseline skeletal survey, with plain radiographs of the skull, ribs, pelvis, spine, and long bones, should be performed in all patients at diagnosis, with most experts recommending a yearly skeletal survey in patients with active disease. For patients suspected of having active disease, magnetic resonance imaging of the spine at diagnosis is strongly suggested. However, magnetic resonance imaging is generally mandated for patients with significant back pain to rule out cord compromise [37]. The role of positron emission tomography is not well established, but some investigators have reported the test's usefulness in patients with extramedullary and bone plasmacytomas [38]. Radionuclide bone scans are generally not useful because the lesions commonly seen in myeloma patients are purely osteolytic and do not generate significant uptake on bone scans [39]. 

 In addition to optimal medical pain control, nonsurgical management of myeloma bone disease includes the use of the antiresorptive bisphosphonates, physical therapy, rehabilitation exercises, as well as judicious use of radiation therapy. 

Bisphosphonates have been shown to inhibit the proliferation and differentiation of osteoclasts with resultant osteoclast apoptosis [40]. Although preclinical studies have suggested an antineoplastic effect of bisphosphonates, this has not been substantiated in clinical studies. A large double-blind randomized study demonstrated that pain and bone-related complications were reduced and performance status and quality of life were improved in myeloma patients with lytic lesions treated with monthly intravenous pamidronate [41, 42]. Zoledronic acid, which has been found to be equally efficacious as pamidronate, can be administered over a shorter period of time than that for pamidronate [42]. Although these agents are generally well tolerated, side effects of therapy occasionally limit their use. Acute complications include flu-like symptoms (related to the first infusion), renal toxicity (with a decrease in incidence with longer infusion duration), and osteonecrosis of the jaw, which occurs in approximately 5% of multiple myeloma patients on chronic monthly bisphosphonate therapy. The incidence of osteonecrosis of the jaw can be decreased with attention to dental hygiene, by delaying therapy until after certain dental procedures, and with less frequent bisphosphonate administration [43, 44]. An American Society of Clinical Oncology expert panel recommends that myeloma patients who have at least one lytic lesion be treated with intravenous pamidronate (90 mg over 2 hours) or zoledronic acid (4 mg over 15–30 minutes) every 3-4 weeks for the first 12 months. Thereafter, therapy should be individualized based on the patient's risk of a skeletal event [45]. Another antiresorptive therapy involving the inhibition of RANKL by a monoclonal antibody (denosumab) is currently the subject of clinical investigations [46]. In addition, novel myeloma therapies, including lenalidomide and bortezomib, have been demonstrated to result in beneficial effects on bone metabolism in multiple myeloma patients [47, 48]. 

 Multiple myeloma cells are exquisitely radiosensitive, and radiation therapy is effective in the treatment of localized bone complications, especially in cases of epidural disease or cord compression. Concerns over compromised marrow reserve and the availability of novel and more effective therapies limit the use of radiation therapy. 

 Little is known about the effect of an exercise program on patients with multiple myeloma. Although a retrospective study suggested that exercise had a positive effect on quality of life [49], bias is likely with such studies, and the type, intensity, and duration of the optimal exercise program have yet to be defined. In our experience, we have anecdotally noted aquatic therapy to be effective in improving mobility while being well tolerated by myeloma patients. Despite occasional reports noting the effectiveness of acupuncture in patients with uncontrolled pain, controlled trials have not been performed, and the use of complementary and alternative medicine cannot be routinely recommended [50].

Surgical intervention is at times necessary for the prevention and treatment of fractures. More recently, patients with painful vertebral compression fractures can be treated with vertebroplasty or balloon kyphoplasty. Vertebroplasty involves injection of poly-methylmethacrylate bone cement in the vertebral body. In one study, pain control was improved in 97% of the patients with the use of this procedure. The incidence of serious complications (such as cement leak) ranges from 1%–3% [51, 52]. Balloon kyphoplasty involves the insertion of an inflatable balloon in the vertebral body, which is then filled with bone cement. In addition to pain control, height restoration can be achieved with this procedure, and in some centers this procedure is preferred over vertebroplasty [53, 54]. Unanswered clinical problems include the utility and timing of intervention in patients with asymptomatic or minimally symptomatic vertebral fractures. 

 Lytic lesions involving weight-bearing long bones may occasionally require prophylactic surgical fixation with intraosseous rods and/or pins. The location of the lytic lesion, degree of cortical destruction, and the impact on patients' mobility and limb function are all taken into consideration for guiding the appropriate surgical intervention [55]. In general, we recommend surgical evaluation by an oncologic orthopedic surgeon for patients who have lytic lesions exhibiting greater than 50% cortical destruction of a weight-bearing long bone or pain with weight bearing.

## 5. Thromboembolic Events

Patients with monoclonal gammopathy of undetermined significance and multiple myeloma are at increased risk of venous thromboembolic events with a baseline prevalence of approximately 10% [56]. Patients with newly diagnosed multiple myeloma are at higher risk of thromboembolic events than patients with relapsed multiple myeloma, possibly a reflection of increased burden of disease. In addition, immunomodulatory drugs (lenalidomide and thalidomide) have been linked to increased thromboembolic events, especially in combination with high-dose corticosteroids and anthracycline chemotherapy [57–59]. Vascular access devices, fractures or immobilization, and decreased performance status all contribute to this increased rate of venous thromboembolic events. The coagulopathy associated with multiple myeloma and monoclonal gammopathy remains poorly understood. Some investigators have suggested that acquired protein C resistance [60], increased factor VIII levels, and abnormal platelet aggregation studies [57] may be contributory; however, the precise mechanism and contribution of each of these findings remain unclear. 

For all multiple myeloma patients receiving therapy with immunomodulatory agents (lenalidomide and thalidomide), primary prophylaxis for venous thromboembolic events is recommended. The prophylactic strategy of choice is often based on the clinical situation and the presence of additional thrombophilic risk factors [61]. Recently, Palumbo et al. [61] randomized newly diagnosed multiple myeloma patients receiving thalidomide-based induction to low-dose aspirin (100 mg daily), prophylactic low molecular weight heparin (Enoxaparin 40 mg SC daily), or fixed-dose warfarin (1.25 mg daily orally) [62]. This group found that rates of venous thromboembolic events and clinically significant bleeding were low overall with all three strategies. We recommend the use of low-dose aspirin in low-risk patients treated with immunomodulatory agents due to cost and ease of administration concerns [62].

Patients who develop a venous thromboembolic event while receiving therapy with immunomodulatory drugs will require therapeutic anticoagulation with low molecular weight heparin or unfractionated heparin followed by warfarin. Therapy with the immunomodulatory drug can be resumed once the patient has been fully anticoagulated and usually should be continued for as long as the patient continues on the offending agent. Retrospective data suggest that these patients do not have increased mortality compared with patients who did not develop a venous thromboembolic event [58].

## 6. Management of Neuropathy

Patients with multiple myeloma develop peripheral neuropathy as a result of the disease itself, secondary to therapeutic agents (bortezomib, thalidomide, vincristine), and secondary to metabolic/nutritional deficiencies (e.g., cobalamin deficiency) [63]. Myeloma-related neuronal injury can be secondary to neuronal infiltration (as in amyloid deposition or light chain deposition disease), with neuronal compression causing radiculopathy, hyperviscosity, cryoglobulinemia, uremia, and autoimmune pathology [64]. Bortezomib-related peripheral neuropathy typically occurs during therapy but has also been noted to occur or worsen soon after discontinuation of therapy [65]. In addition, autonomic neuropathy has been reported in this setting, which then represents a therapeutic challenge. Neuropathy from bortezomib is frequently painful, resulting in dose modifications and interruption of therapy in many patients. Guidelines for bortezomib dose modifications due to peripheral neuropathy have been recently reviewed [66]. Most patients in a recent large randomized trial treated with bortezomib-based therapy experienced improvement of neuropathy on average 1.9 months after discontinuing therapy, and 60% of patients had resolution of symptoms on average 5.9 months later [29]. Thalidomide-induced peripheral neuropathy is often related to the total cumulative dose of the drug; after 12 months of thalidomide therapy, about 70% of patients will develop neuropathy. Unlike bortezomib, the majority of these patients develop grade 1-2 neuropathy versus grade 3-4 in the bortezomib patients, and the thalidomide-induced neuropathy is often permanent [67]. Clinical symptoms include predominantly sensory manifestations such as parestheses and hyperesthesia. Patients may also develop autonomic dysfunction and motor symptoms. Elderly patients are more prone to the development of neurotoxic effects [62]. Lenalidomide, on the other hand, is considered less neurotoxic than thalidomide and has not been reported to result in severe neuropathy [68]. More recently, newer proteasome inhibitors, such as carfilzomib, have been shown to be less neurotoxic than bortezomib [69].

Often, the first step in the evaluation of patients with neuropathy is to exclude urgent potentially reversible etiologies (such as cord compression). In addition, low cobalamin levels have been frequently observed in patients with monoclonal gammopathy and multiple myeloma, and this etiology should be readily identified and treated [70]. For patients who have drug-induced neuropathy, early identification and dose modification are necessary. Myeloma-related neuropathy may improve with therapy, although it seldom resolves. The mainstay of symptomatic treatment has been extrapolated from the treatment used in diabetic patients, which includes antidepressants (amitriptyline, duloxetine), antiepileptics (carbamazepine, gabapentin, pregabalin), and opiates for pain control [71, 72]. In addition, experts have suggested anecdotal benefits with the use of dietary supplements and high-dose vitamins and amino acids (such as L-glutamine, carnitine, coenzyme Q), but these approaches have not been formally evaluated in this setting. 

 Neuropathy in patients with multiple myeloma can result in significant disability and impairment in quality of life. Early identification and management can often spare patients severe toxicity and dysfunction.

 In conclusion, multiple myeloma is a complex disease having a number of complications and therapeutic challenges. The cure for this disease remains evasive. Although novel therapeutic strategies have resulted in improved patient survival, supportive therapy continues to be an essential component in treating complications of this disease. Therefore, further research to evaluate and improve upon existing supportive therapies in multiple myeloma is needed, as this is an important adjunct in the everyday care of patients with this disease.
